# Functional Characterisation of the WW Minimal Domain for Delivering Therapeutic Proteins by Adenovirus Dodecahedron

**DOI:** 10.1371/journal.pone.0045416

**Published:** 2012-09-27

**Authors:** Ana Villegas-Méndez, Pascal Fender, Marina I. Garin, Romy Rothe, Lavinia Liguori, Bruno Marques, Jean-Luc Lenormand

**Affiliations:** 1 HumProTher Laboratory, TheREx, TIMC-IMAG Laboratory, CNRS UMR5525, University Joseph Fourier, La Tronche, France; 2 UVHCI (UMI3265, CNRS/UJF/EMBL), Grenoble, France; 3 Hematopoiesis and Gene Therapy Division, CIEMAT/CIBER of Rare Diseases, Madrid, Spain; Institute of Molecular and Cell Biology, Singapore

## Abstract

Protein transduction offers a great therapeutic potential by efficient delivery of biologically active cargo into cells. The Adenovirus Dd (Dodecahedron) has recently been shown to deliver proteins fused to the tandem WW_2-3-4_ structural domains from the E3 ubiquitin ligase Nedd4. In this study, we conclusively show that Dd is able to efficiently deliver cargo inside living cells, which mainly localize in fast moving endocytic vesicles, supporting active transport along the cytoskeleton. We further improve this delivery system by expressing a panel of 13 WW-GFP mutant forms to characterize their binding properties towards Dd. We identified the domain WW_3_ and its mutant form WW_3__10_13 to be sufficient for optimal binding to Dd. We greatly minimise the interacting WW modules from 20 to 6 kDa without compromising its efficient delivery by Dd. Using these minimal WW domains fused to the tumor suppressor p53 protein, we show efficient cellular uptake and distribution into cancer cells, leading to specific induction of apoptosis in these cells. Taken together, these findings represent a step further towards the development of a Dd-based delivery system for future therapeutic application.

## Introduction

**Figure 1:**
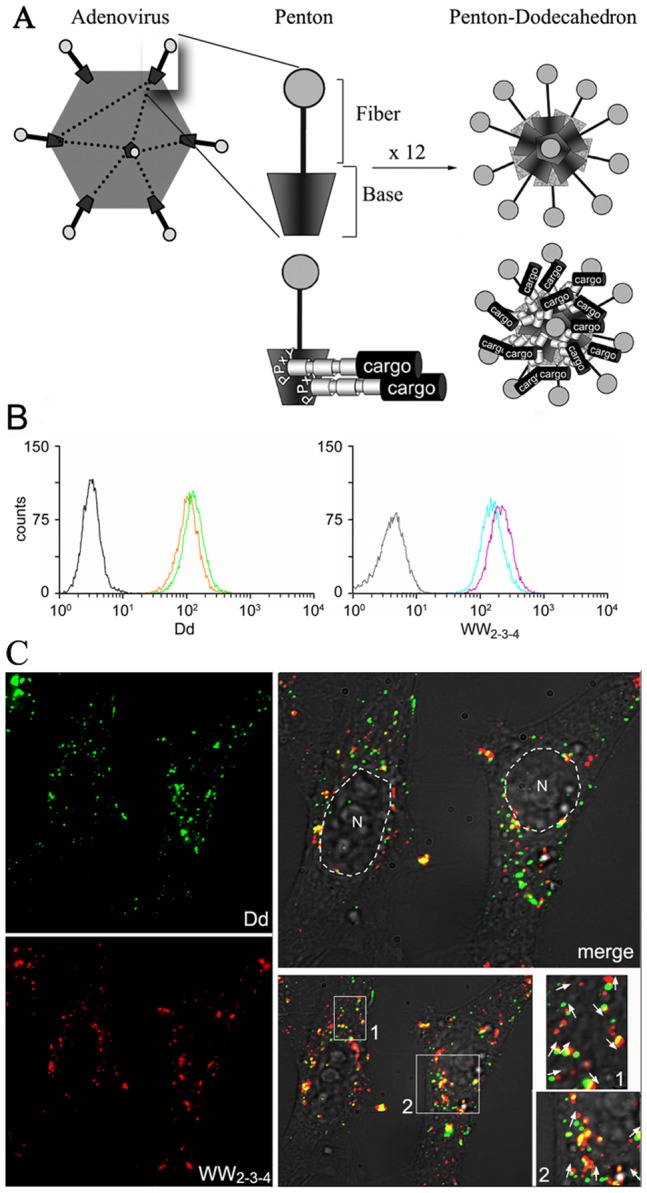
Structure of Penton-Dodecahedron as protein delivery particle and cellular uptake of WW_2-3-4_/Pt-Dd protein complexes in live HeLa cells. *A*. The Adenovirus type 3 Penton structure is a non covalent complex consisting of the fiber and base protein. The conserved PPxY sequences present in the pentameric base region of Pt-Dd serve as docking regions to bind the structurally conserved WW domains from some ubiquitin ligase proteins. *B*. Internalisation of Cy3-Pt-Dd (left panel) and Alexa 647- WW_2-3-4_ (right panel) in cells measured by FACS analysis. Cells were incubated for 2 h with 1.35 nM (orange histogram), 2.7 nM (green histogram) Cy3-Pt-Dd or 0.1 µM Alexa 647- WW_2-3-4_ [internalised by 0.6 nM (cyan histogram) or 1.2 nM (magenta histogram) Pt-Dd]. Non-treated cells, black histogram; cells incubated with Alexa 647- WW_2-3-4_ only,grey histogram. *C.* Internalisation of Cy3-Pt-Dd (signal pseudo-coloured in green for colocalisation purposes) and Alexa 647- WW_2-3-4_ (red) in cells measured by fluorescence microscopy. Cells were incubated with 2.7 nM Cy3-Pt-Dd and 0.3µM Alexa 647- WW_2-3-4_ for 30 min, washed and further incubated with prewarmed media for 3 h before image acquisition using an Olympus Microscope at a rate of 3 frames per min. Frozen images from the live imaging acquisition (see Movie S1) showing the internalisation and cellular distribution of Cy3-Pt-Dd (signal pseudo-coloured in green for colocalization purposes), Alexa 647- WW_2-3-4_ (red signal) and their merged signals and DIC channel extracted from one picture of the Movie S1. Nucleus (N) highlighted in white. Vesicle motion during the Olympus filter switch (about 1 s) is highlighted with white arrows in zoomed areas 1 and 2; areas of Dd and WW_2-3-4_ colocalization are seen in yellow.

Protein therapeutics has recently attracted considerable attention due to its important application in medical treatments. Great efforts are currently focused in the development of innovative delivery systems for therapeutic macromolecules, including proteins, to ensure their stability and specific release into diseased tissue. The capsids of non-enveloped viruses, including Adenovirus (Ad), have evolved exquisite internalization properties suitable for therapeutic application. Although recombinant Ad is one of the most efficient delivery vehicles for gene therapy, the strong cellular and humoral immune response elicited by Ad gene transfer [1], together with the potential risk of harboring viral coding sequences make them unsafe for therapeutic applications. A sub-viral particle from Ad serotype 3 (Ad3) has been proposed as an attractive alternative to Ad for delivery purposes, as (i) it cannot provoke infection given its lack of viral genetic information [2] and (ii) can be easily produce at high scale in a baculorivus system [2]. Ad3 penton base is over-expressed during the viral cell cycle [3], with the ability to self-assemble into dodecahedric particles with fiber proteins protruding from outside, known as Penton-Dodecahedron (Pt-Dd). Interestingly, expression of the base and fiber proteins in a baculovirus system results in the formation of the virus-like particles (VLP) Pt-Dd (Figure 1A). Pt-Dd VLPs are known to efficiently enter a wide variety of cell types [2], [4], [5] and are capable of delivering DNA, chemical compounds or proteins directly into cells [2], [4], [6]–[8]. In addition, we have recently shown that antigen delivery by Pt-Dd can elicit specific anti-tumor immunity in mice bearing B16-OVA tumors [9].

The mechanism of Pt-Dd transduction is, however, somehow different from classical protein transduction domains (PTDs). The fiber knob of Pt-Dd binds to the recently identified primary high-affinity receptor Desmoglein-2 (DSG-2) on the cell surface and triggers transient opening of intercellular junctions, improving access to other receptors [10], [11], [12]. In addition, the high affinity binding of the penton base to heparan sulfate proteoglycans (HSPGs) concentrates Pt-Dd on the cell surface, which favours interaction of the penton base RGD motif with αvβ3 and αvβ5 integrins for efficient endocytic uptake [5], [13], [14].

The Ad3 penton proteins contain two strictly conserved N-terminus PPxY motifs, which are involved in the interaction with WW domains of Nedd4 (neural precursor cell expressed, developmentally down-regulated 4), which belongs to a family of E3 ubiquitin-protein ligases [15], [16]. The binding of the penton to WW structural domains from Nedd4 can be exploited to deliver proteins with therapeutic potential into target cells, by engineering fusion partners to WW. Given that Pt-Dd contains 12 pentameric bases, 60 tandems of WW-interacting PPxY motifs are potentially accessible for cargo attachment, constituting a highly efficient delivery system estimated to internalise 2×10^7^ molecules per cell [4].

Despite the great cellular internalisation observed in transduction experiments using Pt-Dd to internalise proteins fused to WW domains [4], the uptake mechanisms in living cells has not been investigated to date. Moreover, WW domains are present in some cellular proteins and their delivery along the cargo could have a deleterious effect upon cargo functionality or its *in vivo* stability. It is therefore advisable to reduce the size of the WW domains while retaining the Pt-Dd delivery properties. To address these questions, we have performed further biochemical characterisation of Pt-Dd delivery of WW-fusion proteins. Using live-imaging techniques and FACS analysis, we demonstrate the feasibility of Pt-Dd to efficiently deliver cargo inside living cells. By rational design of WW constructs based on sequence alignment of WW containing proteins and analysis of their binding properties to Pt-Dd in an ELISA-based assay, we greatly minimise the size of the interacting WW modules without compromising its delivery by Pt-Dd. Importantly, fusion of the tumour suppresor p53 protein to WW domains for Pt-Dd delivery induces apoptosis in cancer cells to a greater degree than chemotherapy drugs. Taken together, our data demonstrates that the Ad3 derived VLP Pt-Dd serves as a powerful delivery vector with therapeutic application to treat human malignancies.

## Results

### Pt-Dd can Deliver Proteins in Live Cells and Colocalize in Endocytic Vesicles

Previous studies have shown the ability of Pt-Dd to internalize macromolecules including WW-fusion proteins [4] and live imaging microscopy experiments demonstrate that the internalized Pt-Dd colocalizes with endosome markers [9]. We wanted to conclusively demonstrate that the internalization of proteins by Pt-Dd is not due to any artifactual uptake by cell fixation or as a result of cell membrane adherence [17], [18]. To address this point, we incubated HeLa cells with labelled Cy3-Pt-Dd or Pt-Dd preincubated with labelled Alexa 647 WW_2-3-4_. Internalization of Dd and WW_2-3-4_ was analyzed by FACS after trypsin treatment. The internalization of Cy3-Pt-Dd in live cells is observed as a shift of fluorescence in the cell population exposed to either 2.5 µg or 5 µg Cy3-Pt-Dd (Figure 1B left panel, orange and green histograms, respectively) as compared to the non-treated cells (Figure 1B left panel, black histogram). As shown in Figure 1B, right panel, 100% of the cells incubated with 0.1 µM of Alexa 647 WW_2-3-4_ and either 0.75 µg (cyan histogram) or 1.5 µg (magenta histogram) of Pt-Dd show a clear shift of fluorescence, indicating an efficient internalization of the labelled WW_2-3-4_. Comparison to cells treated with Alexa 647 WW_2-3-4_ protein in the absence of Pt-Dd (Figure 1B right panel, grey histogram) demonstrates that WW_2-3-4_ protein transduction in live cells is mediated by Pt-Dd. We then investigated the cellular internalization and trafficking of Alexa 647 WW_2-3-4_ and Cy3-Pt-Dd by live imaging microscopy. Both labelled protein complexes were incubated with HeLa cells and their internalization followed in real time (Figure 1C and Movie S1). Similar to the internalization observed by FACS analysis, all the cells are stained with both Cy3 and Alexa 647 signals (Figure 1C), demonstrating the high internalization efficiency of both Pt-Dd and WW_2-3-4_ protein. This signal is mainly vesicular with larger vesicles observed at the outer nuclear periphery, in good correlation with previous work where Pt-Dd internalization was found to follow an endocytic pathway and accumulate at the nuclear membrane [2]. Fast vesicles motion are observed with both Cy3 (Pt-Dd) and Alexa 647(WW_2-3-4_) channels (see Movie S1). This fast motility makes difficult the colocalization analysis as vesicles moved during the lag of fluorescence filters swap (about 1 second). However, in pictures extracted from the Movie S1, colocalization is clearly seen in slow moving vesicles as yellow signal (Figure 1C, zoomed areas 1 and 2). Moreover, by taking into account the acquisition lag between green and red signal it is possible to extrapolate vesicle motion direction (Fig. 1C, arrows indicate vesicle direction).

### Binding of Multiple WW Domains to Pt-Dd is Mainly Mediated by WW_3_ Domain

**Figure 2:**
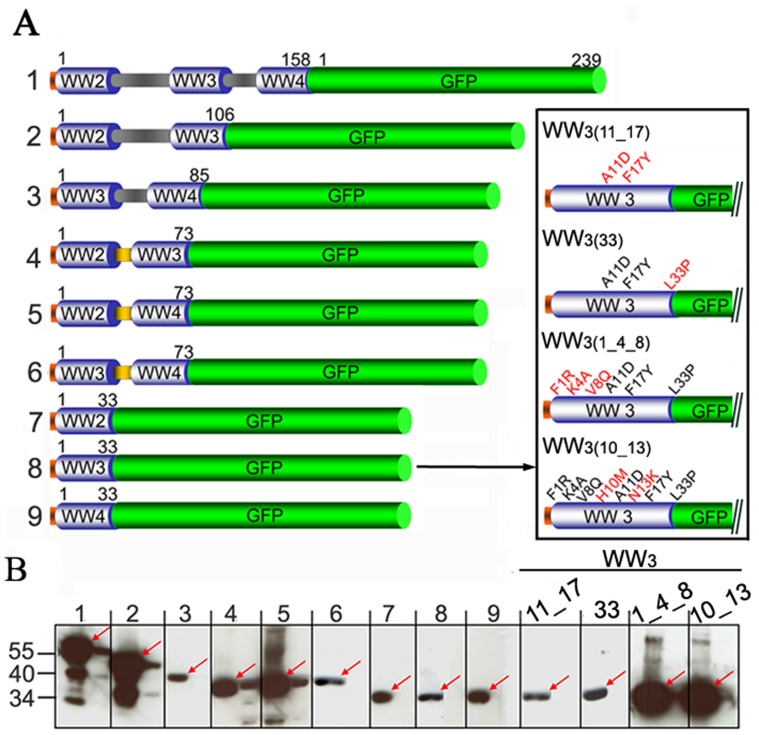
Design of WW fusion proteins and their expression. *A*. GFP fusion constructs were cloned in frame with WW domains from the ubiquitin-ligase Nedd4, including truncated forms containing the shorter linker QGLQNEE (depicted in yellow) and the mutant forms (magnified insert; sequential mutated residues highlighted in red) derived from WW_3_ domain, to generate a closely related form to the artificial WW domain CC43 (1). 6-amino acid histidine tag (orange); WW domains (blue barrels); GFP (green barrel). Amino acid length for each domain is indicated. *B.* WW-GFP fusion proteins were expressed in a cell-free expression system overnight at 20°C and their solubility assessed by Western blot analysis, detected with an anti-histidine HRP antibody. Soluble fraction (red arrows), left; insoluble fraction, right.

Given the high efficiency in delivering WW proteins by Pt-Dd, we can envisage that fusion partners to WW modules would be equally internalized. However, refinement of the binding domain would be advisable to minimise the Pt-Dd attachment module. This could contribute to achieve maximum therapeutic potential and reduce its potential side effects and immunogenicity. Sequence alignment of WW domains from different E3 ubiquitin ligases (Figure S1A) reveals the presence of two highly conserved tryptophans and an invariant proline [19], [20]. These domains independently adopt a curved three-stranded β-sheet configuration and serve as protein interaction modules that bind to proline-containing target sequences [18]. Based on these structural requirements, we designed GFP-fusion constructs to different WW combinations from Nedd4. Sequence analysis of the connecting loops between WW domains from Nedd4, AIP4 and WWP1 revealed that while WW_2_ and WW_3_ domains are interspaced by 40 to 47 amino acids in all the proteins, WW_3_ and WW_4_ domains in AIP4 a WWP1 are separated by a conserved stretch of 7 amino acids (QGQLNEK and QGLQNEE, respectively) instead of the 19 amino acids present in Nedd4 (Figure S1B). In order to minimize the size of the constructs comprising two WW domains (Figure 2A, constructs 2 and 3), we substituted the natural linker regions of Nedd4 by QGLQNEE (in orange, Figure 2A, constructs 4 to 6). In addition to studying the contribution from each individual WW module upon Pt-Dd binding (Figure 2A, constructs 7 to 9), we also generated the mutant forms WW_3__11_17, WW_3__33, WW_3__1_4_8 and WW_3__10_13 by rounds of site-directed mutagenesis (Figure 2A, amino acids highlighted in red). The amino acid substitutions F1R/K4A/V8Q/H10M/A11D/N13K/F17Y/L33P were introduced to generate a closely related form to the artificial WW domain CC43. This CC43 domain, originally created through statistical coupling analysis-based protein design [21], displays enhanced binding properties towards PPxY sequences [22] and could therefore constitute a good candidate as binding module to Pt-Dd.

**Figure 3:**
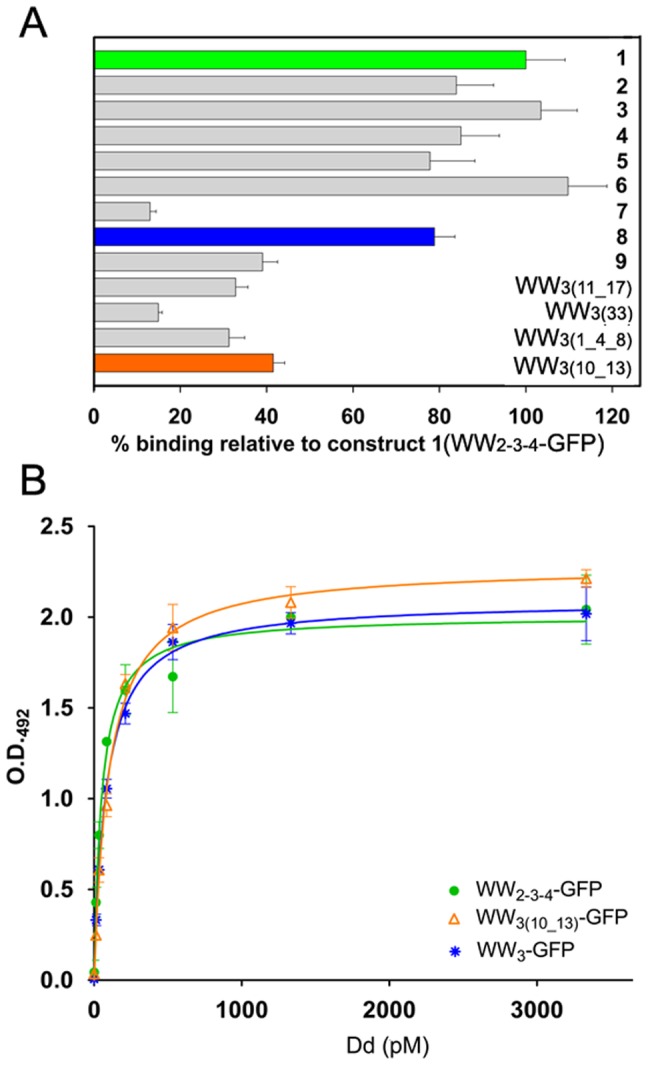
ELISA-based binding assay of WW-GFP and Pt-Dd. WW-GFP proteins were immobilised on 96-well plates coated with anti-GFP antibody. Increasing concentrations of Pt-Dd (0–3.33 nM) were allowed to interact with captured WW-GFP as described in Materials and Methods. *A.* Percentage of relative Pt-Dd binding of WW-GFP constructs to WW_2-3-4_-GFP (construct 1). The data represent the mean ± SD of *n* = 3. *B.* Binding curves of WW_2-3-4_-GFP (green circles), WW_3_-GFP (blue asterisks) and WW_3__10_13-GFP (orange triangles) to Pt-Dd. Values from the ELISA assay were fitted to a non-linear one-site ligand binding equation (GraphPad Prism software) to attribute K_D(app)_ (apparent equilibrium dissociation constants).

All GFP-fusion proteins were expressed as His_8_-tagged proteins in cell-free protein expression system and subjected to Western blot analysis to evaluate their expression levels and solubility (Figure 2B and Figure S2). Analysis of the soluble and insoluble fractions revealed that all the GFP-fusion proteins were expressed in their soluble forms (red arrows), especially with high yields for constructs 1, 2, WW_3__1_4_8 and WW_3__10_13. The binding properties of each GFP-fusion protein towards Pt-Dd were determined using an ELISA binding assay. The soluble fractions from the cell-free reactions were directly used to immobilise WW-GFP fusion proteins by capture to an anti-GFP antibody onto a microtiter plate and bound Pt-Dd detected with anti-Dd and HRP coupled antibodies. Figure 3A shows the percentage of Pt-Dd binding for each different WW-GFP protein relative to WW_2-3-4_-GFP (construct 1, green bar), based on their K_D(app)_. Binding of WW_2-3-4_-GFP to Pt-Dd was found to be of high affinity nature, with an estimated K_D(app)_ of 52.5±11.7 pM (n = 9 from 3 independent experiments, see Figure 3B). Removal of WW_2_ does not have any effect upon binding, as proteins containing WW_3_ and WW_4_ bind equally to Pt-Dd (constructs 3 and 6). A slight decrease in affinity (∼ 20%) is observed with proteins presenting WW_2_–WW_3_ or WW_2_–WW_ 4_ (constructs 2, 4 and 5). Interestingly, WW_3_ (construct 8, blue bar) appears to be the module with highest affinity for Pt-Dd, contributing to 80% of binding as compared to WW_2_ (construct 7, 13% binding) or WW_4_ (construct 9, 40% binding). However, introduction of sequential mutations in WW_3_ does not improve binding but decreases it by approximately threefold, except for the mutant WW_3__10_13 (orange bar), where only twofold decrease in binding is observed. Therefore, although WW_3_ retains good binding properties towards Pt-Dd, it seems that interaction is favoured especially when WW_3_ and WW_4_ modules are present, which corroborates previous observations suggesting cooperative effect between domains [4]. Nevertheless in order to reduce the size of the adaptor domain, we selected WW_3_-GFP and WW_3__10_13-GFP as candidate proteins for Dd delivery into cells, as they bind to Pt-Dd with a K_D(app)_ of 82.4±4.3 pM and 100±6.4 pM, respectively (n = 3), similar to WW_2-3-4_-GFP (Figure 3B).

### WW_3_-GFP and WW_3__10_13-GFP can be Delivered to Cells by Pt-Dd with Similar Efficiency as WW_2-3-4_-GFP

**Figure 4:**
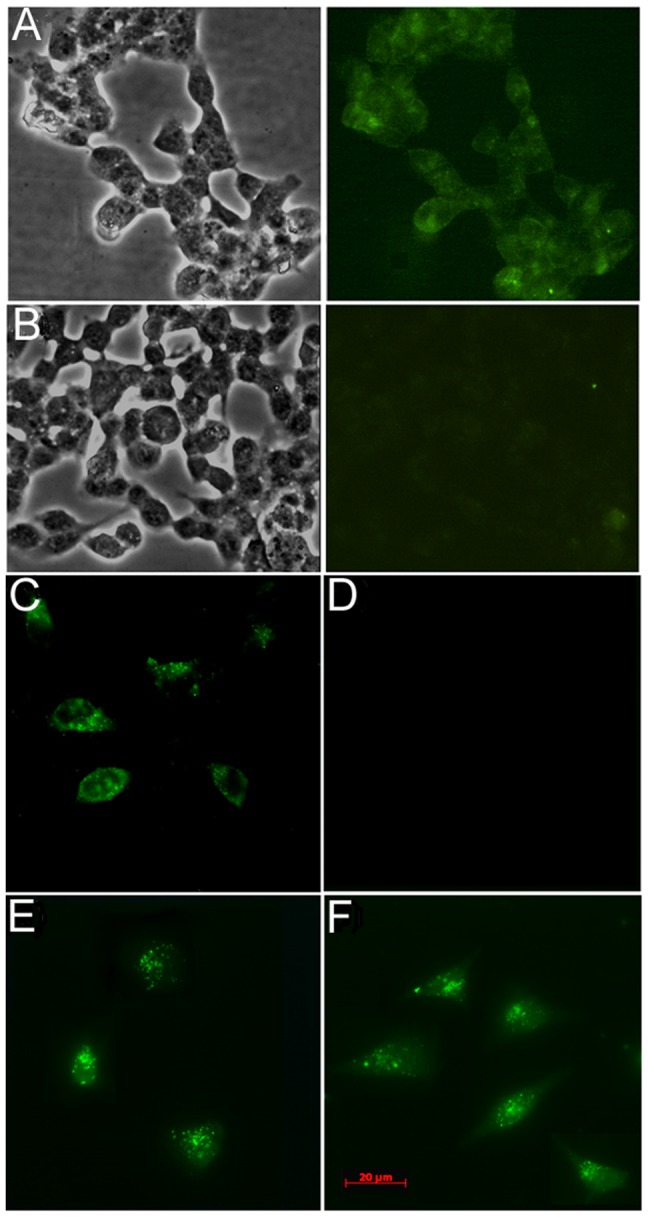
Cellular internalization of WW-GFP by Dd. WW-GFP proteins or GFP were incubated with Pt-Dd to allow protein complex formation and incubated with HCT116 (*A,B*) or HeLa (*C–F*) cells. Protein internalization was visualized by inverted fluorescence microscopy on live cells directly (*A,B*) or fixed cells with anti-GFP antibody (*C–F*). WW_2-3-4_-GFP (*A,C*); GFP (*B,D*); WW_3_-GFP (*E*); WW_3__10_13-GFP (*F*).

To investigate the ability of Pt-Dd to deliver cargo fused to WW domains, we incubated cells with Pt-Dd/WW-GFP fusion proteins complexes and monitor their uptake by microscopy analysis. First, we studied the internalization of WW_2-3-4_-GFP by direct visualization in live cells (Figure 4A). Similar to Alexa 647 WW_2–4_ uptake observed by flow cytometry and live imaging, Pt-Dd was able to deliver GFP inside the cells, with a punctuated signal characteristic of Pt-Dd entry into cells. The internalization of GFP was directly mediated by the interaction of Pt-Dd with WW_2-3-4_ (Figure 4A), since GFP alone was not internalized by Pt-Dd (Figure 4B). Although the internalization of WW_2-3-4_-GFP can be appreciated widely distributed as punctuated signals in the cytoplasm of 100% of the cells, the signal was weak and bleached rapidly at long exposure times. However, detection of the GFP by immunofluorescence using a specific anti-GFP antibody demonstrates the efficient uptake of WW_2-3-4_-GFP by Pt-Dd (Figure 4C) and not GFP alone (Figure 4D). A similar pattern of internalization is observed when cells are incubated with Pt-Dd and WW_3_-GFP or WW_3__10_13-GFP (Figure 4E and 4F, respectively). This result corroborates the ability of Pt-Dd to efficiently interact with WW_3_ domain of Nedd4 (either natural or mutant form closest to the synthetic CC43) and deliver the fused cargo inside cells.

### WW_2-3-4_-p53^wt^ Retains the Ability to Bind p53 Sequence-specific DNA Sequences and Induces Apoptosis in Cancer Cells

**Figure 5:**
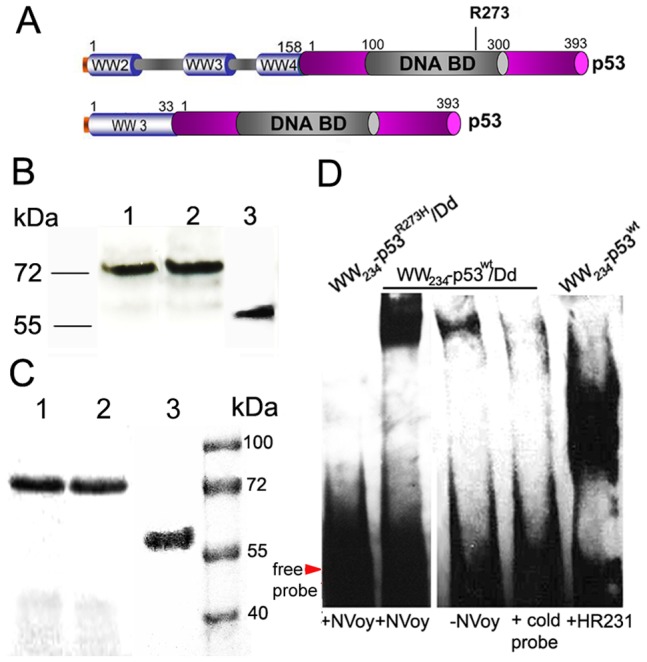
Expression of WW-p53 fusion proteins and DNA binding activity. *A.* Schematic representation of WW-p53 fusion proteins. The cDNAs from human p53^wt^ and a p53^R273H^ mutant form were cloned in frame with the WW domains from Nedd4. WW_2-3-4_ or WW_3_ domains, blue barrels; p53, violet barrel; DNA binding domain, grey insert. Numbers indicate protein amino acid length. *B.* WW-p53 fusion proteins were expressed in *E coli* BL21 and the soluble fraction separated by SDS-PAGE followed by Western blot using an anti-histidine HRP antibody. *C*. Proteins were purified by nickel affinity chromatography and analyzed on SDS-PAGE gels by Coomassie Blue staining. Lane 1, WW_2-3-4_- p53^wt^; lane 2, WW_2-3-4_- p53^R273H^; lane 3, WW_3_- p53^wt^. *D.* The DNA binding activity of WW_2-3-4_- p53^wt^ in complex to Pt-Dd was measured by EMSA, as described in the Material and Methods section, using a p53 specific DNA probe and detected by chemiluminescence.

We previously showed that delivery of ovalbumin as model antigen by Pt-Dd results in a specific anti-tumor immunity in mice bearing B16-OVA tumors [9]. Here, we determined whether Pt-Dd is capable to deliver p53^wt^ protein for inducing apoptosis in tumor cells. We designed and generated recombinant proteins including p53^wt^ and p53^R273H^ mutant (defective in the transactivation) fused to either WW_2-3-4_ (WW_2-3-4_-p53^wt^ and WW_2-3-4_-p53^R273H^) or WW_3_ (WW_3_- p53^wt^) and WW_3__10_13 (WW_3__10_13 - p53^wt^) to evaluate the capability of Pt-Dd to mediate their uptake into cells (Figure 5A). All p53 fusion proteins (with the exception of WW_3__10_13 - p53^wt^) were correctly expressed as soluble proteins in BL21 *E.coli* cells (Figure 5B, lanes 1 and 2). We included the NVoy molecule during purification of p53, as it is an unstable transcription factor which easily precipitates during the purification steps. NVoy is an amphipathic linear carbohydrate-based polymer which associates with surface-exposed hydrophobic patches, stabilising proteins by prevention of aggregation and non-specific binding. As shown in Figure 5C, soluble p53 proteins were purified near homogeneity by affinity chromatography. First, we evaluated whether the DNA binding property of p53 proteins was preserved when fused to the WW domains (WW_2-3-4_ and WW_3_) and in the presence or absence of Pt-Dd and NVoy. As shown in Figure 5D, WW_2-3-4_-p53^wt^ protein is able to bind to specific p53 target sequences in the presence of Pt-Dd, whereas its mutant form p53^R273H^ fails to recognise the probe. The specificity of the binding is corroborated by preincubation of WW_2-3-4_-p53^wt^ protein with the anti-p53 antibody HR231. Interestingly, addition of NVoy greatly improves binding of WW-p53 to the p53 DNA specific probe, suggesting this carbohydrate stabilises the fusion protein.

**Figure 6:**
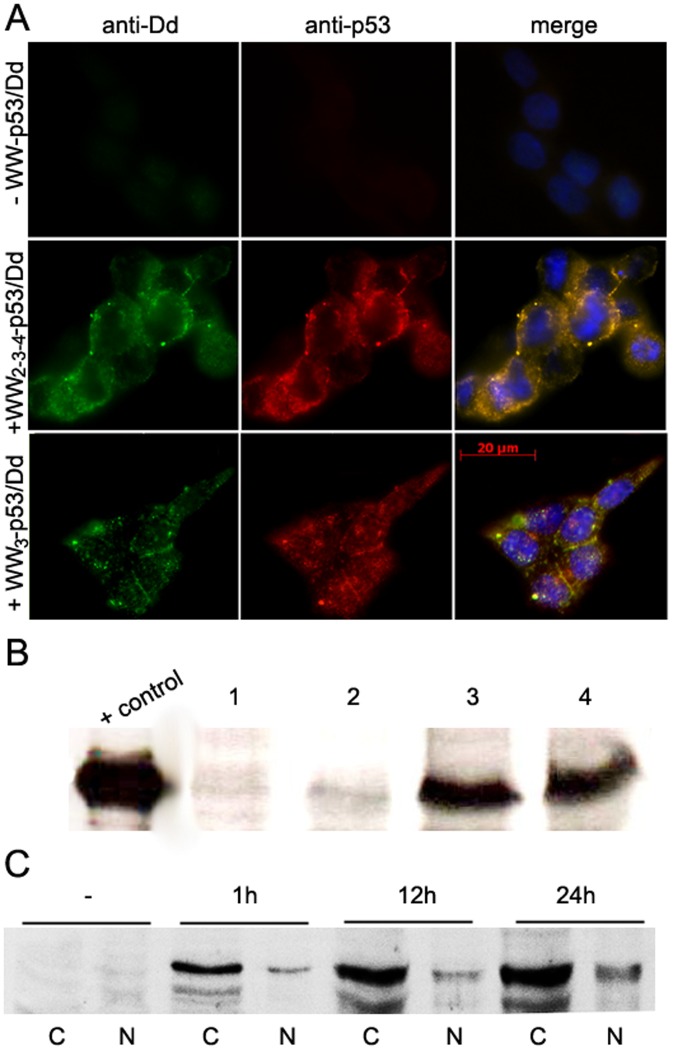
Pt-Dd mediated uptake of WW-p53 fusion proteins into HCT p53^−/−^ cells. *A.* Cells were incubated with 0.2 µM WW-p53/Pt-Dd complexes for an hour and internalized proteins detected by immunocytochemistry using anti-Dd and anti-p53 antibodies. Merge panels show the co-localization of WW-p53 and Pt-Dd inside the cells. *B, C*. HCT p53^−/−^ cells were incubated with WW-p53/Dd protein complexes and Dd cell mediated internalization of WW-p53 was analysed by Western blot using an anti-p53 antibody in HCT p53^−/−^ total cell lysates after 2h incubation (*B*) or in HCT p53^−/−^ cytoplasmic (C) and nuclear (N) fractions after a time-course treatment (*C*). Control, purified ww-p53^wt^; 1, non treated cells; 2, cells treated with ww-p53^ wt^ only; 3, cells treated with ww-p53^wt^+ Pt-Dd; 4, cells treated with ww-p53^R273H^+ Pt-Dd.

Once we confirmed the fusion of WW_2-3-4_ to p53^wt^ protein and the presence of Pt-Dd does not affect its binding to p53 consensus sequences, we investigated the ability of Pt-Dd to deliver therapeutic macromolecules fused to WW domains. HCT116 p53−/− cells were incubated with Pt-Dd in the presence or absence of WW_2-3-4_-p53^wt^ or WW_3_- p53^wt^. p53-deficient HCT116 cells failed to induce apoptosis and to sustain an arrest in the G2 phase of the cell cycle after DNA damage [23]. As revealed by immunocytochemistry, Pt-Dd is able to efficiently transduce WW_2-3-4_ or WW_3_ fused to p53^wt^ into HCT116 p53^−/−^ (Figure 6A). Fluorescent secondary antibodies show in the merged images, co-localization inside the cells of both WW-p53^wt^ and Pt-Dd (Figure 6A). We did not observe any signal in the control panels in the absence of the WW-p53^wt^/Dd protein complexes. To ascertain that Pt-Dd mediates the internalization of WW-p53^wt^ or WW-p53^R273H^ mutant into the cells, cells were incubated during 2h with WW-p53^wt^ or WW-p53^R273H^ mutant in the presence or absence of Pt-Dd. After extensive washing and trypsinization, total cell lysates were recovered and the internalization of WW-p53^wt^ or WW-p53^R273H^ mutant proteins was analyzed by western blotting using an anti-p53 antibody. Both exogenous p53 proteins were clearly detected in HCT116 p53−/− cell lysates in the presence of Pt-Dd (Figure 6B, lanes 3 and 4), whereas no signal was detected in non treated cells or in cells treated only with WW-p53 protein (Figure 6B, lanes 1 and 2, respectively). Interestingly, accumulation of the p53 proteins was detectable in both the cytoplasm and the nucleus of the transduced cells after a time-course treatment (Figure 6C), indicating that neither the Pt-Dd nor the presence of WW domain interfere with the cellular localization of the p53 proteins. These results led us to conclude that Dd can transduce WW-p53 protein complexes into the cells with high efficiency.

**Figure 7:**
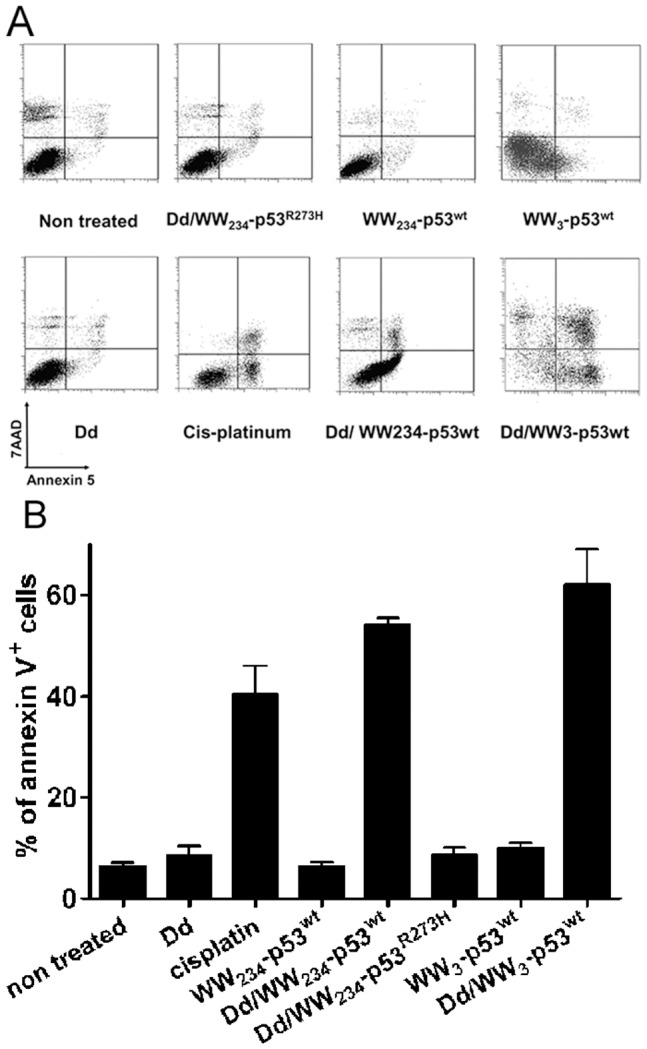
Exogenous WW-p53 internalised by Pt-Dd can induce apoptosis in HCT p53^−/−^ cells. *A.* HCT p53^−/−^ cells were incubated for 1 h with WW-p53 proteins (3 µg), Pt-Dd (2 µg) or WW-p53/Pt-Dd protein complexes and cellular apoptosis was measured by flow cytometry after 36 hours treatment using an annexinV assay. Positive control included treatment with 20 mM cis-platinum. *A*. Representative plots showing Annexin V and 7AAD staining of HCT p53^−/−^ cells after treatments. *B*. Percentage of apoptotic cells as determined by Annexin V+ staining from *A*. Data shown are the mean and SD of two to three independent experiments.

In order to determine whether the exogenous p53 proteins fused to the WW domains retained functionality after transduction into cells, we checked the capability of WW-p53^wt^ or WW-p53^R273H^ mutant to induce apoptosis in HCT116 p53−/− cells (Figure 7). Treatment of HCT116 p53^−/−^ cells with either WW_2-3-4_-p53^wt^ or WW_3_- p53^wt^ protein complexes in the presence of Pt-Dd resulted in a rapid induction of apoptosis as determined by the percentage of Annexin V positive cells (Figure 7A and 7B). Interestingly, treatment of cells with either WW_2-3-4_-p53^wt^ or WW_3_- p53^wt^ in the presence of Pt-Dd resulted in 50 to 60% of Annexin V positive cells whereas WW_2-3-4_-p53^R273H^ mutant in the presence of Pt-Dd did not result in apoptosis (Figure 7A and 7B). Strikingly, the level of apoptosis achieved by the transduced WW_2-3-4_-p53^wt^ or WW_3_- p53^wt^ proteins was greater than those obtained with the drug cisplatin and was not due to the presence of Pt-Dd or NVoy in the protein preparation, as incubation of HCT116 p53^−/−^ cells with Pt-Dd or incubation of cells with WW_2-3-4_-p53^wt^ or WW_3_- p53^wt^ proteins alone failed to induce any apoptosis (Figure 7A and 7B),Consistent with our transduction results mediated by Pt-Dd, p53^wt^ protein fused to the WW domains sustained its pro-apoptotic function after cellular internalization. Taken together, these results demonstrate that Pt-Dd is capable to mediate delivery of active therapeutic proteins fused to either WW_2-3-4_ or the minimal WW_3_ domain and induce apoptosis in cancer cells.

## Discussion

An increasing body of evidence demonstrates that internalization of PTDs is a multi-step process, initiated by strong ionic interactions with HSPGs or other electrostatic interactions with negatively charged moieties on the cell surface, leading to an endocytosis PTD-mediated uptake [17], [18], [24], [25]. Similarly, Pt-Dd strongly interacts through the penton base proteins with HSPGs in the nanomolar range [5], concentrating the particle on the cell surface. The mechanism of Pt-Dd uptake is, however, different from other PTDs, as it specifically binds to DSG-2 as primary high affinity receptor [10], [11], [12] and requires integrins as sole entry receptor [13]. Identification of the Pt-Dd cellular internalization process is crucial to better tailor this sub-viral particle as delivery tool. For therapeutic applications it is equally important to demonstrate its capability to internalize cargo in living cells and exclude any potentially artifactual uptake. In this study, we conclusively show the suitability of Pt-Dd to deliver proteins directly inside living cells as efficiently as reported in previous studies using fixed cells [4], [7]. We also improve the delivery system by elucidation of the minimal WW domain required for cargo attachment to Pt-Dd without compromising its delivery properties. More importantly, Pt-Dd is able to deliver p53 fused to WW domains in a functional state to induce apoptosis of cancer cells.

Our data analysis on the cellular uptake of labelled Cy3-Pt-Dd demonstrates a very efficient delivery system, which reaches almost saturation at 1.35 nM (Figure 1B left panel, orange histogram). Any possible surface-bound Pt-Dd particles were removed by trypsin treatment before analysis to exclude artifactual readings. Pt-Dd was able to internalize the Alexa 647 labelled protein WW_2-3-4_ with high efficiency (Figure 1B, right panel, cyan and magenta histograms), an uptake exclusively mediated by this VLP as addition of Alexa 647 WW_2-3-4_ protein on its own (Figure 1B, right panel, grey histogram) could not be internalized. Moreover, all the PPxY sites in the Pt-Dd (10 per base pentamer, that is 120 PPxY sequences per Pt-Dd particle) seem to be occupied by WW_2-3-4_, as delivery of this protein is almost saturated at a molar ratio of 1∶150 of Pt-Dd to WW_2-3-4_ (Figure 1B, right panel, cyan histogram). These results were corroborated by real-time live imaging microscopy, where both Cy3-Pt-Dd and Alexa 647 WW_2-3-4_ could be observed in vesicular compartments (Figure 1C). This punctuated distribution of the Pt-Dd/WW_2-3-4_ particles suggests endocytic uptake, in agreement with the importance of HSPGs and integrins as receptors involved in attachment and internalisation of Pt-Dd, colocalization with the Rab5 endosomal marker [9] as well as the ATP- and temperature-dependent uptake [5], [13]. A striking finding from the imaging analysis was the fast motion of vesicles containing Pt-Dd and WW_2-3-4_ (see Movie S1), which strongly suggest the involvement of active transport along the cytoskeleton. In support of this hypothesis, Ad capsids are known to interact with microtubules, a process mediated by molecular machines such as cytoplasmic dynein, which drives capsid motility towards the MTOC (microtubule organising center) in the perinuclear region [26]. Similarly, TAT conjugated to quantum dots are actively transported to localise in the MTOC [27] and TAT-peptide internalisation via macropinocytosis also requires actin filaments [28]. Our finding of concentrated Pt-Dd and WW_2-3-4_ vesicles in the outer nuclear periphery could therefore indicate that a similar mechanism of active transport process from the cell periphery to the perinuclear region is taking place.

Endocytosed particles normally traffic from early to late endosomes and lysosomes, characterised by progressive compartment acidification. This is the case for endocytosed TAT peptide, since the fluorescence signal of TAT is greatly decreased when tagged to fluorescein as compared to Alexa Fluor 488 [24]. We observe a similar loss of fluorescence when directly monitoring the internalization of WW_2-3-4_-GFP (Figure 4A) while the signal is not affected when detected with anti-GFP and Alexa Fluor 488 labelled antibodies (Figure 4, panels C, E and F), suggesting a similar entrapment of Pt-Dd/WW-GFP particles in acidic vesicles. Although GFP and some of its variants (including EGFP, used in our study) are more resistant to photobleaching than fluorescein, the fluorescence of EGFP decreases rapidly below pH 7 [29] and this pH sensitivity is similar in intracellular organelles [30]. Trapping of delivered cargo within these acidic compartments could lead to their degradation by proteases, compromising effective delivery of bioactive molecules. Thus, escape of PTDs from endosomal vesicles to cytoplasm is generally accepted as the rate-limiting factor in transduction efficiency. Nevertheless, the biological effects exerted by functionally active proteins both *in vitro* [31]–[34] and *in vivo* [35]–[38] suggest that at least an undetectable fraction of the delivered cargo is released into target organelles. In fact, the bioavailability of functional cargo has been demonstrated by different groups using the more sensitive Cre-mediated recombination reporter assay [39], [40]. To overcome the high dose concentrations of proteins required for effective biological response, the pH-dependent fusogenic peptide HA2 from influenza virus has been proposed as endosome disrupting agent. Despite the initial excitement of markedly enhanced escape of cargo from macropinosomes [25], inconclusive results have been reported by others [39], [41], [42] and its effectiveness *in vivo* has yet to be proven. Although our microscopy data only supports cargo entrapment into vesicles, it could be envisaged that an undetectable amount of protein is released into the cytoplasm. In fact, delivery of p53 by Pt-Dd (Fig. 6) and particularly the induction of apoptosis by this exogenous p53 (Fig. 7) reveals that at least a part of the internalized p53 remained functionally active. Alternatively, Dd penton base could be implicated in the release of cargo from endosomes [43], since this protein is subjected to endosome pH-induced conformational changes leading to membrane disruption [44]. This interaction of the penton base with endosomal membrane could be however hampered by masking of the co-internalised cargo, in a similar way as observed with anti-penton base antibodies, which cause intracellular neutralisation of Ad virions [44]. Reducing the size of the cargo and the WW attachment modules would therefore alleviate the vesicle entrapment. Contrary to subgroup C viruses, membrane lysis for subgroup B capsids (including serotypes 3, 7, 9 and 35) only takes place when they reach compartments that match their optimal pH (that is, late endosomes or lysosomes) and the fiber protein has been proposed to influence this membrane lytic machinery [45], [46]. One alternative could be to swap the fiber in Dd for a subgroup C fiber to favour earlier escape from endosomes, which could translate into an increased bioavailability of functional cargo.

Our binding analysis of Pt-Dd to WW-fusion partners confirm the high affinity interaction of Ad base proteins and WW domains [4], [15]. By ELISA binding assay, we estimated that Pt-Dd binds to WW_2-3-4_-GFP with an affinity in the picomolar range. Previous kinetic analysis of Ad2 penton proteins to the WW domains of Nedd4-like ubiquitin ligase WWP1 yielded an affinity value of 65 nM [15], [16]. This discrepancy in kinetic values could be attributed to differences in binding affinities between Ad2 and Ad3 capsid proteins or between different WW containing proteins. Moreover, the presence of multiple PPxY motifs in the penton base (pentameric structure) and repetition of WW domains could lead to avidity, making this interaction of complex nature and its kinetic analysis in quantitative terms is therefore only approximate. Previous studies in the interaction of Pt-Dd towards MBP (maltose binding protein)-WW fusion proteins demonstrated the binding is saturated at a 2 nM MBP-WW [4], in good correlation with our binding analysis. Despite these caveats in kinetic estimation of the interaction between Pt-Dd and WW-fusion proteins, the data presented here serve as a basis to analyse the contribution of different WW domains towards Pt-Dd binding. We found that constructs containing WW_3_ and WW_4_ present similar affinity to Pt-Dd as WW_2-3-4_. However, the binding is decreased to different degree when only one WW module is present, which corroborates cooperative effects between domains [4]. Similarly to the preferential binding of the WW_3_ from Nedd4 to VP40 of Ebola virus or to its natural target, the epithelial sodium channel [46], [47], we observed that WW_3_ is the predominant domain for Pt-Dd binding. Mutations introduced in this domain to obtain WW_3__10_13-GFP (close to the synthetic WW CC43 [20], [22], which displays a 10-fold increase binding compared to its natural counterpart) do not improve binding in our analysis. It has to be noted, however, that the binding affinity of WW_3__10_13-GFP to Pt-Dd is of high affinity nature while CC43 binds to PPxY sequences (as assessed by Trp fluorescence quenching using a PPxY peptide library) in the micromolar range [22]. These differences in binding could arise from a weaker interaction between peptides as opposed to the whole interacting partner (in this case, Pt-Dd) and the reduction to a 1∶1 stechiometry. Despite the complexity of the kinetic analysis, our binding studies allowed us to select the minimal WW domain constructs that form stable complexes with Pt-Dd. Most importantly, Pt-Dd is able to internalize the selected constructs WW_3_-GFP and WW_3__10_13-GFP into cells (Figure 4E and F) with similar efficiency as WW_2-3-4_-GFP (Figure 4C).

Here, we used p53-deficient human colon carcinoma HCT116 cells [23] to validate the capability of the Pt-Dd system to deliver bioactive full length proteins. The tumour suppresor p53 protein is a crucial transcription factor that orchestrates the response to DNA damage or deregulation of mitogenic oncogenes, by direct induction of protein expression involved in cell-cycle arrest or by triggering apoptosis or cellular senescence if the damage is severe, ultimately restricting proliferation (reviewed in [49]). Mutations in the p53 gene is one of the most frequent genetic alterations in about 50% of all cancers, resulting in dysfunction of the p53 protein leading to tumour progression and genetic instability. In addition, tumours with wild-type p53 often carry mutations in other genes involved in the regulation of p53 protein. The p53 protein is therefore an attractive candidate for cancer therapy and recent studies demonstrate that its reactivation or overexpression lead universally to tumour regression of established tumours [50]–[52]. We provided experimental evidences that p53^wt^ protein fused to the WW domains and carried by the Pt-Dd still retains its function after cellular uptake. We showed that WW_2-3-4_-p53^wt^ or WW_3_- p53^wt^ proteins treatment induced significant apoptosis in HCT116 p53^−/−^ cells. Intracellular localisation of exogenous p53 proteins showed accumulation of the proteins into the nucleus and cytoplasm, indicating that Pt-Dd is a very high efficient system for delivering active therapeutic macromolecules inside the cells. Furthermore, neither Pt-Dd nor WW domains interfere with the cellular distribution and function of the p53 proteins. This is quite a different process compared to that used by the L domain of some retroviral Gag proteins which is involved in the recruitment of cellular WW containing proteins. As previously described, this protein recruitment can interfere with cellular functions for stimulating the budding of the virus [55], [56]. Additionally, we cannot exclude that once released from the endosomal vesicles, either the Pt-Dd or the mutated WW domain from the recombinant protein may interact with some host cellular proteins resulting in a modification of some cellular pathways. Different therapeutic approaches have been tested for rescuing p53 function in tumour cells [53], [54]. These strategies are mainly based on small molecules capable to either stabilize the folding of mutant p53 in tumour cells or by preventing the inhibition of MDM2 factor to wild-type p53 [53]. Nevertheless, none of these strategies are fully effective for treating cancers because they only focused on some p53 functions. Consequently, our results using p53 full-length protein which encompasses the entire cellular functions and Pt-Dd as a delivery system suggest that this approach may potentially represent a powerful therapeutic strategy for treating cancers.

Adenoviral-based cargo delivery can be regarded as a versatile delivery tool. Pt-Dd could be exploited for the delivery of apoptotic proteins or drugs for the treatment of malignancies of epithelial origin, where its primary high-affinity receptor DSG-2 is overexpressed [10]. To mention, the Pt-Dd structure could be further improved for delivery purposes by specific targeting to different cellular receptors through modification of cell-recognition domains in the fiber [57] or the addition of monoclonal antibodies to target overexpressed receptors in tumors, such as herceptin or erbitux to target Her2/neu or EGFR in breast and colon cancer, respectively. Pt-Dd offers the additional advantage of making some receptors, including Her/neu, more accessible to the cell surface by transient opening of intercellular junctions [10]. Additionally, Dd cellular tropism and the fate of delivery could be modified by creating chimeric Dd from different Ad serotypes [45], [46]. In conclusion, the work presented here demonstrates the feasibility of Pt-Dd to internalise cargo with high efficiency in live cells. This VLP delivery system was optimised by greatly minimising the WW attachment module without impairing its endocytosis uptake, which constitutes a step further in the development of Pt-Dd for therapeutic applications.

## Materials and Methods

### Generation of WW Expression Constructs and Mutagenesis

The cDNA of WW_2-3-4_ domains from human ubiquitin ligase Nedd4 were cloned into pET15bΔt- WW_2-3-4_ expression vector as previously described [9] and used as template to generate WW_2-3-4_ truncated forms by standard PCR techniques. Additional constructs were also designed to substitute the Nedd4 natural linker region between domains WW_2_-WW_3_ and WW_3_-WW_4_ by the shorter WW conserved sequence 5′-CAGGGTCTGCAGAACGAAGAA-3′ (coding for amino acids QGLQNEE). WW domains including this sequence were amplified by PCR using specific primers and first cloned into pET30b before subcloning into pET15bΔt. WW_3_ mutants were generated by sequential rounds of amino acid substitutions using the QuikChange™ Site-Directed Mutagenesis Kit following the manufacturer’s instructions (Stratagene). Those included mutant WW_3(11_17)_ (substitutions A11D and F17Y**)**, mutant WW_3(33)_ (substitutions A11D, F17Y and L33P), mutant WW_3(1_4_8)_ (substitutions F1R, K4A, V8Q, A11D, F17Y and L33P) and mutant WW_3(10_13)_ (substitutions F1R, K4A, V8Q, H10M, A11D, N13K, F17Y and L33P). To generate WW-GFP fusion proteins, EGFP was amplified by PCR using peGFP plasmid as template (Invitrogen) and cloned into pET15bΔt WW vectors. Human p53^wt^ and p53^R273H^ mutant form are cloned in frame with WW_2-3-4_ pET15bΔt vector to generate WW_2-3-4_-p53^wt^ and WW_2-3-4_-p53^R273H^ fusion proteins. p53^wt^ was also cloned in frame with domains WW_3_ and WW_3(10_13)_.

### Protein Expression and Purification

A protein expression screening and solubility test for all WW-EGFP fusion proteins was first performed, using the RTS *E.Coli* HY 100 cell-free expression system (Roche Applied Science) overnight at 20°C. For protein scale up, WW_2-3-4_ protein and WW_2-3-4_-EGFP, WW_3_-EGFP and WW_3(10_13)_-EGFP fusion proteins were expressed in *E.coli* strain BL21 (DE3) (Novagen) and purified by affinity chromatography methods as previously described [9]. WW-p53 fusion proteins were expressed in BL21 by induction with 0.1 mM IPTG overnight at 20°C. To purify the recombinant proteins, BL21 cells were lysed by sonication in binding buffer [25 mM Tris pH 8, 150 mM NaCl, 5 mM imidazole, 2 mM DTT, 15% glycerol and protease inhibitors (pepstatin, E-64, aprotinin, Pefabloc, and complete protease inhibitor mixture; Roche Applied Science]. Cleared lysates were incubated with Ni^2+^ beads (Promega) containing Nvoy for 3 h at 4°C. Beads were washed sequentially with binding buffer containing 500 mM NaCl and 10–60 mM imidazole and proteins were eluted with elution buffer (binding buffer containing 500 mM imidazole and NVoy). Proteins were PBS buffer exchanged by ultrafiltration in Vivaspin 2 columns (Sartorious) and stored at −80°C until used. Pt-Dd was prepared using the baculovirus expression system as previously described [2], [14]. Protein solubility were assessed by Western blot analysis with an anti-histidine HRP antibody (Sigma-Aldrich). Protein concentration and purity was assessed by SDS-PAGE and stained with PageBlue™ (Fermentas).

### ELISA-based Binding Assay

All binding experiments were investigated by ELISA-based binding assay using a capture approach. First, Immuno 96 MaxiSorp™ plates (Nalge Nunc International) were coated overnight at 4°C with 0.3 µg/well of a purified goat anti-GFP IgG (Rockland Immunochemicals) diluted in coating buffer (0.1 M Na_2_CO_3_ pH 9.6). Unreacted sites were blocked with 250 µl/well of 3% BSA in PBS for 6 h at 37°C. The soluble fractions from cell-free reactions expressing each WW-GFP construct were diluted in washing buffer (1% BSA, 0.05% Tween 20 in PBS), ranging from 1∶40 to 1∶300 dilution (according to protein expression levels) and 100 µl/well incubated for 1 h at 37°C with gentle shaking. Excess of ligand was removed by washing four times with washing buffer. Increasing amounts of purified Pt-Dd (0–200 ng/ml in washing buffer) were added to each well and incubated for 1 h at 37°C. After washing, bound Pt-Dd to WW-GFP fusion proteins was detected with 100 µl/well of rabbit anti-Pt-Dd antibody diluted 1/100,000 and 100 µl/well of donkey anti-rabbit-HRP diluted 1/10,000 (GE Healthcare). Bound HRP antibody was detected with SIGMA*FAST*™ OPD substrate (Sigma-Aldrich). The reaction was terminated by addition of 50 µl of 3 M H_2_SO_4_ solution. Absorbance from each binding reaction was measured at O.D._492 nm_ using a LB 941 Tristar microplate reader (Berthold Technologies). Values were fitted to a non-linear one-site ligand binding equation (GraphPad Prism software) to attribute K_D(app)_ (apparent equilibrium dissociation constant) for each WW-GFP Pt-Dd interaction.

### Electrophoretic Mobility Shift Assay (EMSA)

The DNA-binding activity of WW_2-3-4_-p53^wt^ protein was assayed by EMSA. A p53 sequence-specific DNA probe was made by annealing oligonucleotides 5′-AAT GTC CGG GCA TGT CCG GGC ATG TCC GGG CAT GT-3′ (Forward) and 5′-AAT CAT GCC CGG ACA TGC CCG GAC ATG CCC GGA CA-3′ (Reverse). The annealed probe was labelled using Biotin dUTP and purified on a G-25 spin column (Active Motif). 500 ng-1.5 µg of WW_2-3-4_-p53^wt^ or WW_2-3-4_-p53^R273H^ protein was incubated with 1–3 µg of Pt-Dd for 30 min and equilibrated for further 30 min at RT with 4x binding buffer B-2 and stabilizing buffer (Active Motif). 0.5 pmoles of biotinilated probe was mixed with binding buffer C2 and stabilizing buffer and incubated with samples for 1 h at RT. Control samples included a competition assay by excess of cold probe and WW_2-3-4_-p53^wt^ supershift with anti-human p53 monoclonal antibody (kind gift of Pr Thierry Soussi (Department of Oncology-Pathology, Cancer Center Karolinska (CCK), Karolinska Institute in Stockholm, Sweden) HR231. The DNA-protein complexes were separated on 4% native polyacrylamide gels in 0.5x Tris borate/EDTA and transferred to a Hybond N+ nylon membrane (GE Healthcare). DNA-protein bands were detected using the LightShift® Chemiluminescent EMSA Kit (Pierce), following the manufacturer’s instructions.

### Cell Cultures

HCT116 p53^−/−^ colon carcinoma (described in [23], a gift from B. Vogelstein) and HeLa cells were maintained in McCoy’s 5A medium containing 40 µg/ml G418 (Invitrogen) or DMEM medium with GlutaMAX™ (Invitrogen), respectively. Culture media were supplemented with 10% FCS (Invitrogen), 50 units/ml penicillin and 50 ug/ml streptomycin (Invitrogen).

### Flow Cytometry Analysis of Protein Internalization

HeLa cells were seeded on 12-well plates at 1×10^5^ cells/well and cultured for 24 h. WW_2-3-4_ and Pt-Dd were fluorescently labelled by coupling it to Alexa 647 (Molecular Probes) and Cy3 dyes (GE Healthcare), respectively, following the manufacturer’s instructions. 0.75 µg Alexa 647-WW_2-3-4_ was incubated with either 0.75 µg or 1.5 µg Pt-Dd for 30 minutes. Samples were added to 250 µl of supplemented DMEM medium and incubated with HeLa cells for 2 h. Control experiments included treatment with Cy3-Pt-Dd and Alexa 647-WW_2-3-4_ separately. After treatment, cells were harvested by trypsinization and resuspended in PBS. Internalized proteins were monitored by flow cytometry on a FACSCalibur (BD Biosciences) and analysed using CellQuest software.

### Real-time Microscopy of Protein Internalization

HeLa cells were seeded at 5×10^4^ on a 24-well glass dish and cultured overnight. 2 µg of Cy3-Pt-Dd was incubated with 2 µg of Alexa 647-WW_2-3-4_ for 30 minutes. Samples were added to 200 µl of cold EMEM medium and incubated with HeLa cells for 30 minutes at 4°C. Cells were washed and further incubated for 3 h with 200 µl prewarmed DMEM-10%FCS. Acquisition was performed at 3 frames per minute in a thermostated chamber connected to an Olympus IX81 inverted Microscope, using the DIC, Cy3 and Fast-TexRed channels with the 60X objective. Cy3 signal was pseudo-coloured in green. Pictures were extracted using Volocity software.

### Fluorescence Microscopy and Immunocytochemistry

HCT116 p53^−/−^ and HeLa cells were seeded at 2 × 10^4^ on 8-well Lab-Tek™ chamber slides (Thermo Fisher Scientific) and cultured overnight. 0.8–1.5 µg of the indicated WW-EGFP or WW-p53 fusion proteins were incubated with 0.8 µg Pt-Dd for 30 min. Control experiments included incubation of cells with medium only or with Pt-Dd and EGFP. Samples were added to 100 µl of supplemented DMEM or McCoy’s 5A medium and incubated with cells for 1 h. For protein internalization experiments on live cells, chamber slides were washed three times with PBS before visualization. Immunofluorescence studies were performed as described previously [9]. EGFP was detected with anti-eGFP diluted 1∶1000 (Euromedex) and secondary Alexa 488 anti-mouse antibody diluted 1∶1000 (Molecular probes). For co-localization studies, p53 was detected with anti-human p53 DO-7 clone diluted 1∶100 (BD Biosciences) and Pt-Dd with rabbit anti-Pt-Dd sera diluted 1∶1000 [3]. Primary antibodies to p53 and Pt-Dd were detected with Alexa Fluor 546 anti-mouse antibody and Alexa Fluor 488 anti-rabbit antibody, respectively (Molecular probes), diluted 1∶1000. Nuclei were counter stained with Hoechst 33258 (Molecular probes) and slides mounted with mounting medium (Dako). Internalized proteins were visualised using a Nikon Eclipse TE 2000 inverted fluorescence microscopy.

### Western Blot Analysis of Internalised Proteins

HCT116 p53^−/−^ cells were grown in 6-well plates until they reached 60–80% confluency. Cells were washed twice with PBS and incubated for 2 h with 0.2 µM WW-p53/Pt-Dd protein complexes, washed thrice with PBS and lysed in RIPA buffer (50 mM Tris, pH 7.4, 150 mM NaCl, 1% NP-40, 0.1% SDS and 1x complete protease inhibitor cocktail). For time-course analysis of internalized WW-p53, cytoplasmic and nuclear fractions were prepared after 1 h, 12 h and 24 h of addition of WW-p53/Pt-Dd protein complexes using the compartmental protein extraction kit (Chemicon®, Millipore). A total of 50–100 µg of whole cell extracts or cellular fractions were subjected to SDS-PAGE and transferred to nitrocellulose membranes. WW-p53 proteins were detected by Western blot using the anti-p53 antibody (clone DO-7) diluted 1∶500 and secondary HRP-labelled anti-mouse antibody diluted 1∶5000 (Amersham Biosciences).

### Apoptosis of HCT116 p53^−/−^ Cells After ww-p53/Pt-Dd Treatment

HCT116 p53^−/−^ cells were seeded on 24-well plates until they reached 60–80% confluency. Cells were washed twice with PBS and incubated with WW-p53 proteins (3 µg), Pt-Dd (2 µg) or WW-p53/Pt-Dd protein complexes for 1 h. Positive control experiments included treatment with the chemotherapeutic drug cis-platinum (Sigma-Aldrich) at 20 µM final concentration. Cellular apoptosis was assessed after 36h treatment by flow cytometry using the Annexin-V-FLUOS Staining kit (Roche Applied Science), following the manufacturer’s recommendations.

## Supporting Information

Figure S1 A. Individual alignment of each WW domains 1 to 4 from NEDD4 (**N**eural precursor cell **E**xpressed, **D**evelopmentally **D**own-regulated **4**), AIP4 (**A**trophin-1 **I**nteracting **P**rotein **4**), WWP1 (**WW** domain-containing **P**rotein **1**) and the artificial WW domain CC43. B. Alignment of the WW domain regions 2 to 4 from NEDD4, AIP4 and WWPI WW_2-3-4_, including their domain connecting loops. Conserved tryptophans are highlighted by asterisks.
(TIF)

Figure S2 **SDS-PAGE analysis of purified proteins.** Nedd4 WW_2-3-4_ (lane 1) and WW-GFP selected fusion constructs (lane 2, construct 8; lane 3, construct WW_3_10_13_; lane 4, construct 1) were expressed in *Escherichia coli* strain BL21, purified from cells supernatants on nickel sepharose HisGraviTrap columns and PBS buffer exchanged by ultrafiltration.
(TIF)

Movie S1 **Real-time cellular uptake of WW_2-3-4_ by Pt-Dd.** Cells were incubated with 2.7 nM Cy3-Pt-Dd and 0.3 µM Alexa 647- WW_2-3-4_ and their internalization followed in real-time using an Olympus Microscope at a rate of 3 frames per min. The live imaging acquisition shows the cellular distribution of the internalized Pt-Dd (pseudo-coloured in green) and WW_2-3-4_ (red signal).
(MOV)
