# Supplementary figures and images for: Functional Characterisation of the WW Minimal Domain for Delivering Therapeutic Proteins by Adenovirus Dodecahedron
Source: PLoS One. 2012 Sep 27;7(9):e45416. doi: 10.1371/journal.pone.0045416 (PMC3459938; doi:10.1371/journal.pone.0045416)

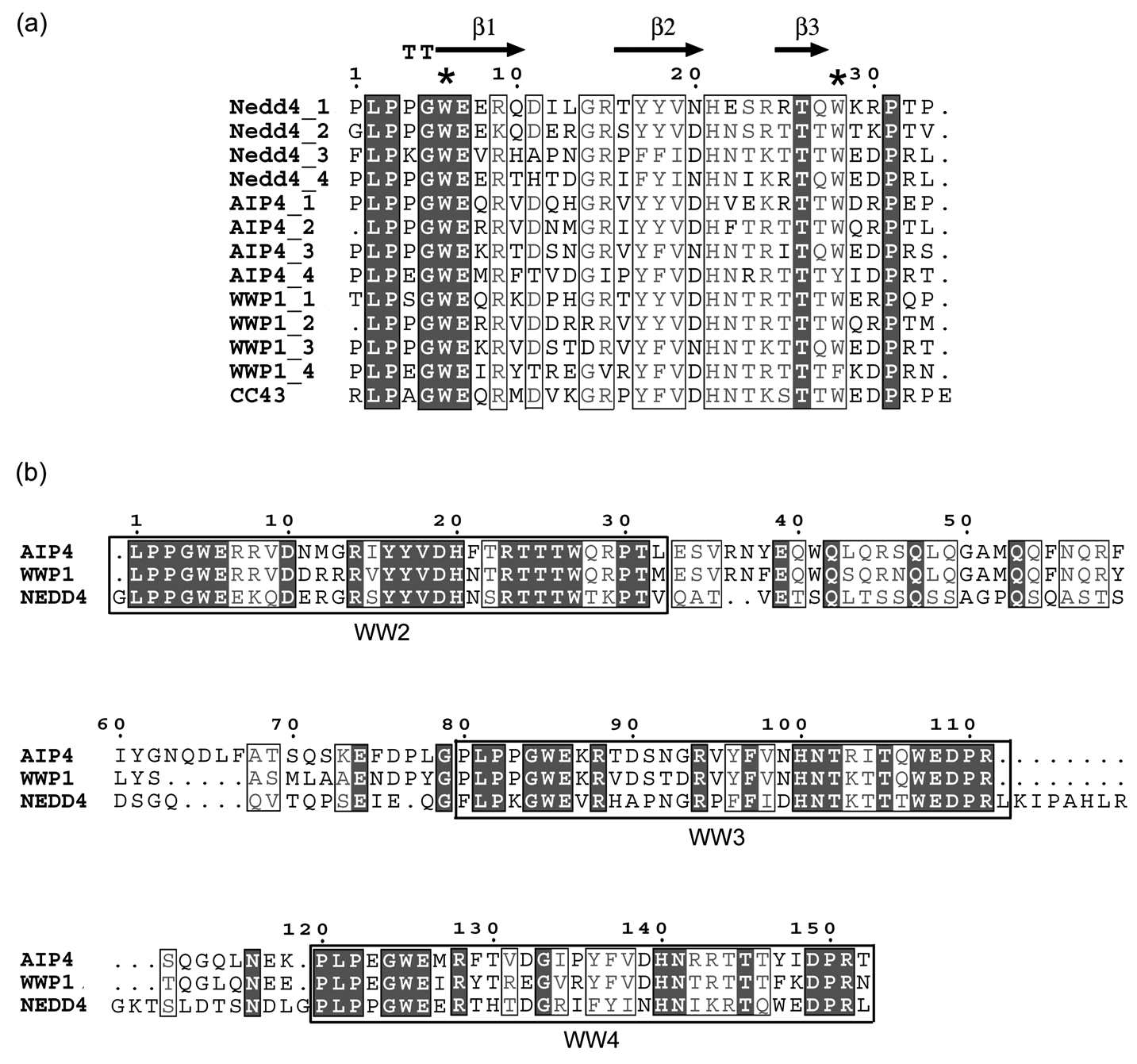

Supplement: Figure S1 — A. Individual alignment of each WW domains 1 to 4 from NEDD4 (Neural precursor cell Expressed, Developmentally Down-regulated 4), AIP4 (Atrophin-1 Interacting Protein 4), WWP1 (WW domain-containing Protein 1) and the artificial WW domain CC43. B. Alignment of the WW domain regions 2 to 4 from NEDD4, AIP4 and WWPI WW2-3-4, including their domain connecting loops. Conserved tryptophans are highlighted by asterisks. (TIF) [file pone.0045416.s001.tif]

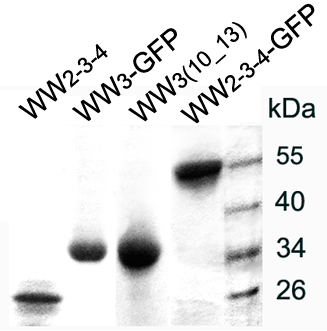

Supplement: Figure S2 — SDS-PAGE analysis of purified proteins. Nedd4 WW2-3-4 (lane 1) and WW-GFP selected fusion constructs (lane 2, construct 8; lane 3, construct WW3_10_13; lane 4, construct 1) were expressed in Escherichia coli strain BL21, purified from cells supernatants on nickel sepharose HisGraviTrap columns and PBS buffer exchanged by ultrafiltration. (TIF) [file pone.0045416.s002.tif]
